# Mixed-phenotype acute leukemia, not otherwise specified, rare types B/T leukemia: a case report in the Jordanian Royal Medical Services

**DOI:** 10.25122/jml-2025-0110

**Published:** 2025-10

**Authors:** Tania Ogeilat, Bayan Al-zghoul, Mohammad Jasser Maaita, Areen Al zghoul, Alaa abu Alkishk, Hind Alqatamin, Mothana Alnawaflh, Falah ALfarajat, Mohammad Masalha, Mousa Qatawneh, Dima Al-dabbas, Hashim Shawabkah, Mohammad Al Qudah, Abdalrahman Mohammad

**Affiliations:** 1 Princess Iman Center for Research and Laboratory Sciences, Jordanian Royal Medical Services, Amman, Jordan; 2 Queen Rania Hospital for Children, Amman, Jordan; 3 Prince Hussein Center for Urology, Amman, Jordan; 4 King Hussein Medical Center, Amman, Jordan

**Keywords:** leukemia, hematology, pediatric

## Abstract

Mixed phenotype acute leukemia (MPAL) represents approximately 3–5% of all acute leukemia cases and is defined by blast populations that co-express markers from more than one hematopoietic lineage. In most cases, blasts exhibit myeloid markers together with either B-cell or T-cell markers. The rarest subtype is mixed B/T acute leukemia. We report the case of a 7-year-old boy who presented with weakness and fatigue and was diagnosed with MPAL, not otherwise specified, B/T rare type, based on bone marrow examination and immunophenotyping. This case highlights the essential role of comprehensive immunophenotyping in establishing an accurate diagnosis of MPAL. Given the limited information in the literature, case series and prospective studies are needed for a better understanding and successful treatment.

## Introduction

Acute leukemia is a type of hematological malignancy characterized by the proliferation of blasts, which normally constitute up to 1% to 5% of bone marrow cells. Based on the antigens expressed by the blasts, their lineage can usually be classified as either myeloid or lymphoid [1]. The classification of acute leukemia is essential for tailoring optimal individual treatment [2]. In a small number of acute leukemia cases, the leukemic cells show no evidence of single lineage differentiation, or they express differentiation antigens highly specific for more than one lineage. These cases, collectively termed acute leukemia of ambiguous lineage, account for <5% of all acute leukemia cases and include both acute undifferentiated leukemia and mixed phenotype acute leukemia (MPAL) [3].

Cases of MPAL usually express a combination of myeloid antigens together with either B-cell or T-cell antigens. The rarest type of MPAL is the combination of B-cell and T-cell components [4]. There are no clear clinical features, genetic anomalies, or prognostic expectations for such cases due to the small number of diagnosed and reported cases in the literature [5].

## Case report

**Figure 1:**
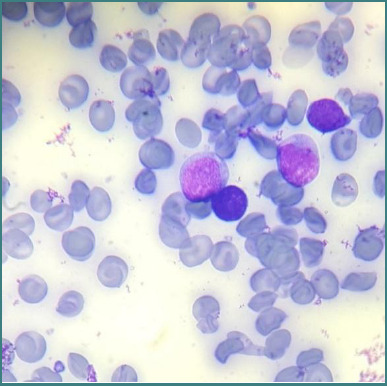
Peripheral blood smear showing circulating blasts

A 7-year-old boy presented to the emergency department of Queen Rania Hospital for Children in 2022, complaining of fatigue and pallor. His laboratory workup revealed a low hemoglobin level of 8.5 g/dL. The total white cell count was 4.2 × 10^3^/ uL, but his absolute neutrophil count and platelet count were low. The patient was admitted to the hospital for further workup. A blood film reported the presence of blasts in the peripheral blood and indicated the need for bone marrow examination and flow cytometry. The bone marrow aspirate was hemodiluted without specules. Smears were stained using May-Grunwald Giemsa stain, and the morphologic examination revealed blasts similar to those seen in the peripheral blood (Figure 1).

**Figure 2:**
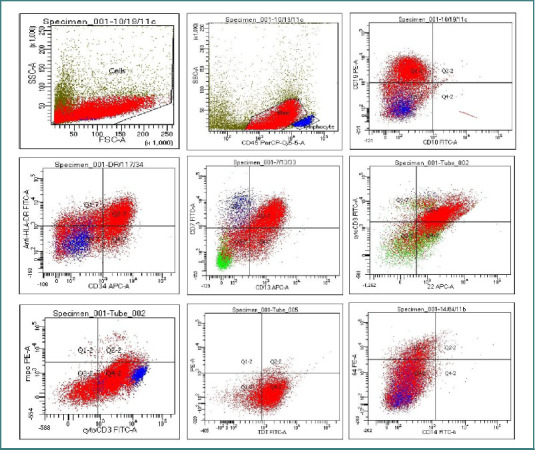
Blasts express CD19, HLA-DR, CD34, CD 7, CD13, cytoplasmic CD3, cytoplasmic CD22, and TdT

Multi-parameter flow cytometry using the Becton Dickinson (BD) fluorescence-activated cell sorter Canto II was performed following the standard operating procedure. The following antigens were assessed: CD34, HLA-DR, TdT, MPO, CD13, CD33, CD11b, CD11c, CD14, CD64, CD117, CD3, CD2, CD5, CD7, CD4, CD8, CD10, CD19, cCD22 and CD79a, which cover the myeloid, B-cell and T-cell lineages. The expression of CD45 was analyzed along with side scatter for blast gating. B-cell and T-cell specific antigens were doubly expressed on the blast population as shown in Figure 2.

The patient was initially started on the Total XIII chemotherapy protocol, but did not achieve remission. He then received two cycles of the FLAG-Ida regimen, after which he achieved complete remission with no detectable minimal residual disease. A haploidentical stem cell transplant from his sister was performed, and full donor chimerism was confirmed. However, 3 months later, disease relapse occurred in the donor-derived bone marrow, as demonstrated by the X/Y FISH study, which showed persistent XX expression. Following reinduction therapy, he achieved a second remission and underwent a second haploidentical transplant. Unfortunately, he experienced another relapse shortly afterward. The patient subsequently developed transplant-related complications, including cytomegalovirus (CMV) infection and Gram-negative bacterial sepsis, and ultimately passed away.

## Discussion

MPAL is a rare type of acute leukemia that is characterized by a bimodal age distribution, predominantly affecting individuals younger than 19 years of age and those over 60 [6]. Our patient, a 7-year-old child, falls within the first peak of this distribution.

Flow cytometry (immunophenotyping) is the gold standard for the evaluation and diagnosis of acute leukemia, as it rapidly detects lineage-specific marker expression on blast populations [7]. Myeloid lineage is characterized by the expression of myeloperoxidase (MPO). B-cell lineage is identified by the coexpression of bright CD19 and CD10 or, when CD10 is absent, by the expression of CD19 together with cytoplasmic CD79a (cCD79a) and CD22. The T-cell component of MPAL is confirmed by strong cytoplasmic CD3 (cCD3) expression [4,5]. All efforts must be made to produce high-quality flow data. This includes obtaining sufficient and representative specimens, gathering enough events (a minimum of 1,000 blasts and 20,000 events per tube), and using an appropriately broad panel of antibodies [2,8]. As mentioned in the previous section, a broad panel was applied for our patient’s diagnosis. The number of acquired events was 30,000.

In order to assert the coexistence of multiple lineages in such cases, careful attention to the positivity threshold is crucial. According to the European Group for the Immunological Classification of Leukemias (EGIL) scoring system, surface antigen expression is considered positive when ≥20% of blasts exceed the control cutoff, while cytoplasmic markers such as myeloperoxidase (MPO), cytoplasmic CD79a (cCD79a), terminal deoxynucleotidyl transferase (TdT), and cytoplasmic CD3 (cCD3) require a ≥10% threshold [9-11]. In our case, 70% of the gated blasts were positive for CD19 and cCD22, which are specific for the B-lineage, as well as cCD3, which defines the T-cell lineage.

Gerr *et al*. recommended initiating treatment with an acute lymphoblastic leukemia (ALL)-directed chemotherapy regimen and escalating to more intensive protocols, including hematopoietic stem cell transplantation (HSCT), in cases of resistance [12]. Our patient was started on the Total XIII ALL protocol, but failed to achieve remission. After that, he received FLAG-Ida, which is an acute myeloid leukemia (AML)-based protocol. This treatment succeeded in inducing remission, but disease relapse occurred after a haploidentical stem cell transplant.

## Conclusion

Similar cases are rarely reported in the literature, and their clinical features and prognostic expectations remain unclear. Comprehensive flow cytometry is critical for diagnosis. Case series and prospective studies focusing on the immunophenotype and the underlying cytogenetic and molecular heterogeneity are essential to optimize individual therapeutic protocols.
